# Conformational Evolution of Bicalutamide in Chloroform: A Comprehensive NMR Study

**DOI:** 10.3390/molecules30224479

**Published:** 2025-11-20

**Authors:** Konstantin V. Belov, Ilya A. Khodov

**Affiliations:** 1 G.A. Krestov Institute of Solution Chemistry of the Russian Academy of Sciences, Ivanovo 153045, Russia; kvb@isc-ras.ru; 2 N.N. Semenov Federal Research Center for Chemical Physics Russian Academy of Sciences, 4 Kosygin Street, Moscow 119991, Russia

**Keywords:** bicalutamide, non-steroidal antiandrogen, conformational polymorphism, solid phase, 2D NOESY

## Abstract

This study presents new findings on the conformational mobility of the nonsteroidal antiandrogen blocker, bicalutamide, in deuterated chloroform. Based on NOE NMR spectral analysis, quantitative information was obtained regarding the distribution of «open» and «closed» conformer groups in four systems: one with a solid phase (16.7%/83.3%), one without the solid phase (19.7%/80.3%), and two diluted solutions at different concentrations (16.1%/83.9% and 85.3%/14.7%). It was shown that the preparation of molecules for nucleation and the transition to the «closed» conformation occurs at low concentrations and is maintained as the concentration increases until the solid phase is formed. This behavior of conformational evolution contrasts previous understandings of the solid phase’s influence on molecular conformation in solution. The results obtained will offer deeper insights into the conformational evolution of small molecules during nucleation, using bicalutamide as a model.

## 1. Introduction

The conformational changes in small molecules in biologically active compounds within solutions have become a key subject of contemporary research [1,2,3,4]. A primary motivation for studying the conformational evolution during desolvation and nucleation is the strong correlation between the conformation of a molecule in a saturated solution and the polymorphic form obtained, particularly in the case of conformation-determined polymorphism [5,6,7,8]. For instance, the polymorphism and solvatomorphism of fluconazole, a flexible molecule, were shown to depend heavily on solvent and desolvation pathways, with careful crystallization techniques allowing for the formation of different polymorphs [9,10]. The ability to manipulate the conformational state of molecules in solution opens up new avenues for developing novel solid forms of active pharmaceutical ingredients [11]. This area of research has gained considerable momentum, particularly with the recent development and integration of the pre-nucleation cluster theory [12,13,14], which provides an alternative to classical nucleation theory. Recent theories, such as pre-nucleation cluster theory, emphasize the role of spatial structure and solvation in nucleation, challenging traditional models that focus on crystallization from a single solute molecule [15]. This new perspective on nucleation suggests significant influence from spatial structure and solvation processes, though the mechanisms behind these effects remain insufficiently understood. Previous studies have identified key factors that affect the predominant conformation of molecules in solution, including the presence or absence of a solid phase [16], state parameters (pressure and temperature) [17,18], and solvent type [19,20].

**Figure 1:**
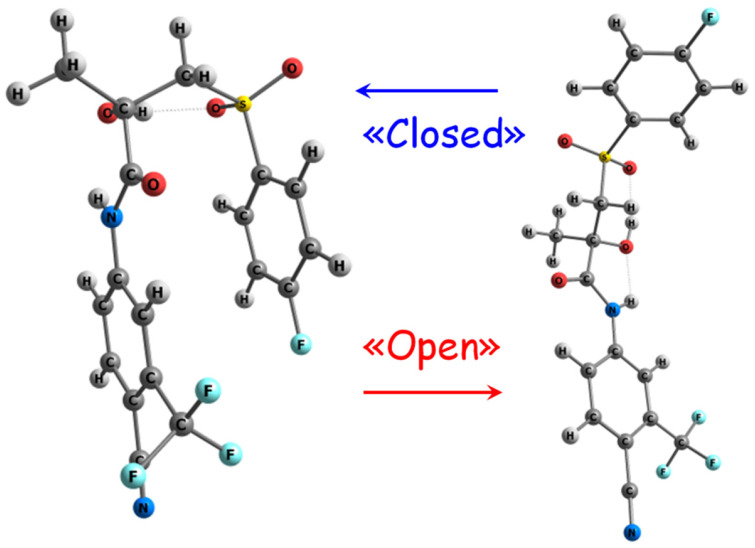
Examples of structures of «closed» (**left**) and «open» (**right**) conformers of BCL.

In the present study, we investigate the conformational evolution of bicalutamide (BCL), a non-steroidal anti-androgenic blocker (testosterone and 5α-dihydrotestosterone), marketed as Casodex^®^, in deuterated chloroform (CDCl_3_) solutions using Overhauser effect nuclear magnetic resonance (NOESY) spectroscopy. BCL exhibits conformation-determined polymorphism and solvatomorphism [21,22,23], with molecules in various crystalline forms existing as «open» and «closed» conformers (see Figure 1), which are characterized and documented in the Cambridge Crystallographic Data Center (CCDC) [23,24,25,26,27].

Despite this, the molecular mechanisms stabilizing these different BCL structures have remained unknown until recently. Our recent study [28] examines BCL conformational polymorphism and solid forms, focusing on how different molecular structures affect stability, solubility, and bioavailability. It explores the role of non-covalent interactions, including hydrogen bonds [29] and π–π stacking [30], in stabilizing polymorphs [7]. Solvent effects, including those from chloroform, are crucial for understanding BCL’s conformational behavior and improving drug formulations. In a series of previous studies, thoroughly outlined in the review [28], a DFT analysis was conducted to optimize and comprehensively characterize the structure and geometric parameters of the «open» and «closed» conformers of BCL, which formed the basis for the present study. However, earlier studies conducted in DMSO did not reveal the concentration-dependent effects of dimerization, as the solution was too supersaturated, and the presence of associates in the solution skewed the results. Further, the high solubility of BCL in DMSO-*d*_6_ allowed us to evaluate the distribution of «open» and «closed» conformers over a wide concentration range (from 0.86 M to 2.16 M). The observed transition from «open» to «closed» conformers, as seen in stable crystalline forms, is consistent with the increasing concentration over time, which leads to the formation of a solid phase. However, with the exceptionally high BCL concentrations in DMSO-*d*_6_ (>150 mg/mL) [31], this system renders quantitative interpretation challenging and does not allow for the selective identification stabilizing factors for the «open» and «closed» structures.

Studying BCL conformers in chloroform, both in the presence and absence of a solid phase (undissolved material), as well as in diluted solutions, is crucial for understanding nucleation processes in compounds with conformational polymorphism. The solid phase influences nucleation and polymorph formation, while without it, BCL molecules transition freely. Solvent conditions and concentration affect conformer dominance, which is crucial for crystallization and controlling polymorph formation in pharmaceuticals [1,18,32]. The results of this work will, for the first time, provide a detailed mechanistic picture of conformational changes in BCL in solutions of varying concentrations, based on experimental data, as well as allow for the clear identification of patterns that stabilize the spatial structure.

## 2. Results and Discussion

**Figure 2:**
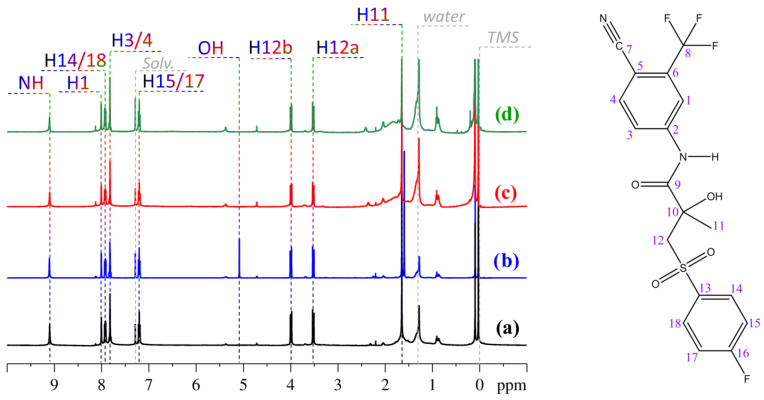
On the (**left**)—1H NMR spectra of BCL in saturated solution (**a**), solution without solid phase (**b**), «high» concentration solution (**c**), and «low» concentration solution (**d**). On the (**right**)—structure of bicalutamide with atom labels corresponding to the signal assignments in the spectra shown.

A comprehensive analysis of the ^1^H NMR spectra for the four studied systems (see Figure 2a–d) was performed, and the resonance signals were assigned based on data from the literature [31]. This allowed for the identification of corresponding hydrogen-containing atom groups in the molecular structure of BCL.

The analysis of the obtained data, compared with the literature, showed that resonance signals corresponding to the para-fluorinated cyclic fragment of the BCL molecules (H14/18, H15/17), as well as the trifluoromethyl-substituted ring (H1, H3/4), and NH groups, appear in the weak-field region. The hydrogen signals from the aliphatic fragment of the molecules predominantly fall in the strong-field region (H12a, H12b, H11). It should be noted that all the studied systems exhibit a water signal originating from the non-anhydrous solvent, as well as a small amount of impurities likely present in the commercial BCL sample. These signals in the ^1^H spectra of BCL have been previously observed when using various solvents [33] and are not discussed further in this work. The OH group hydrogen atom signal is visible in solution of BCL in CDCl_3_ without the solid phase (see Figure 2b). In other cases, this signal was absent due to exchange processes and aggregation at high concentrations [33]. For instance, the OH signal was observed in saturated solution immediately after the sample was prepared and sealed in an ampoule. Experiments were conducted on the same sample after 2, 4, and 18 days, revealing that the OH signal broadens over time (see Figure S1) and becomes indistinguishable at the given scale.

**Figure 3:**
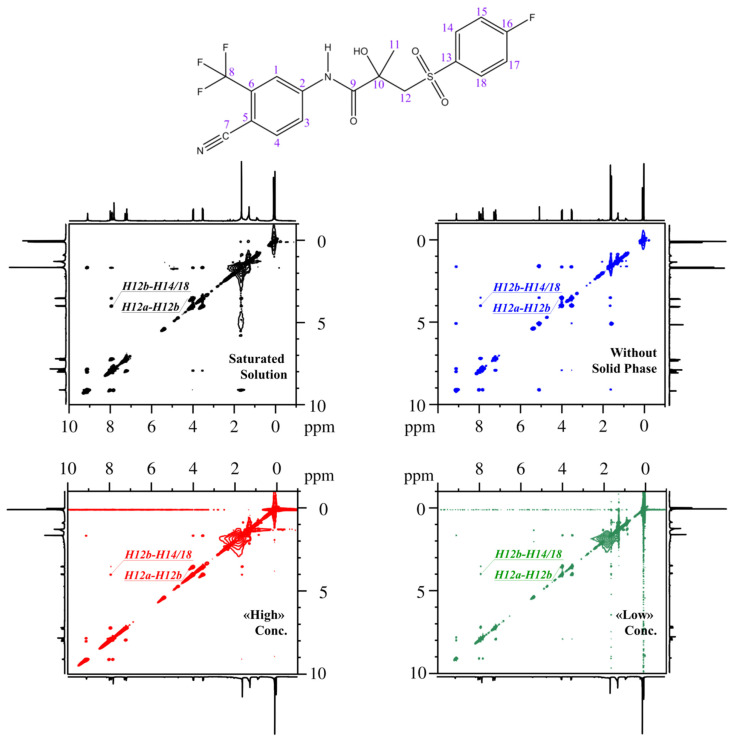
NOESY NMR spectra recorded for a saturated solution (black), solution without the solid phase (blue), solution at «high» concentration (red), and at «low» concentration (green).

The signal assignment data obtained in this study served as the basis for analyzing NOESY spectra and calculating the proportions of «closed» and «open» BCL conformers in CDCl_3_, both in the presence and absence of a solid phase and at various concentrations (see Figure 3). NOESY spectroscopy is a powerful and unique tool for quantitatively analyzing the spatial structure of molecules in solution [34,35,36,37], supercritical fluids [17,38,39,40], biocompatible environments [41,42], and other settings.

The potential of the NOESY method arises from the strong correlation between the cross-relaxation rate (σ, s^−1^)—a parameter directly determined from the experiment—and the corresponding inter-nuclear distance (r, Å)—σ ~ 1/r^6^ [43,44,45]. The value of σ is a weighted average over all conformers in the system—σ = Σσ_i_ × x_i_ [46,47] ]. The value of σ can be obtained within the initial rate approximation (IRA) framework [48,49,50], as the slope of the fitted line in a graph of the averaged integral intensities of cross-peaks [37,51,52,53] as a function of mixing time. By determining the cross-relaxation rates from the NOESY data, the desired inter-nuclear distances can be obtained using the isolated spin pair model (ISPA) [47,54,55,56]—r_exp_ = r_0_ × (σ_0_/σ_exp_)^1/6^ (where r_exp_ and σ_exp_ are the unknown inter-nuclear distance and the corresponding cross-relaxation rate; r_0_ and σ_0_ are the reference distance and the corresponding cross-relaxation rate, which remain constant for all studied conformers) [37,57].

For the proper comparison and interpretation of the data obtained in this work, we used the characteristic conformer-defined distance (r_exp_—H12b-H14/18) and the reference distance (r_0_—H12a-H12b) proposed in a previous study [33]. The averaged values [58] of the conformer-defined distances for «closed» and «open» BCL conformers are: r_Close_ = 4.28 ± 0.18 Å; r_Open_ = 3.46 ± 0.49 Å. The reference distance (r_0_) is 1.78 ± 0.49 Å for all examined conformers. The cross-relaxation rates and experimentally determined H12b-H14/18 distances for the four studied systems are listed in Table 1. The graphs of the averaged integral intensities as a function of mixing time, used for cross-relaxation rate calculations, are shown in Figure S2. The data required to reproduce the experimental results are provided in Table S1.

**Figure 4:**
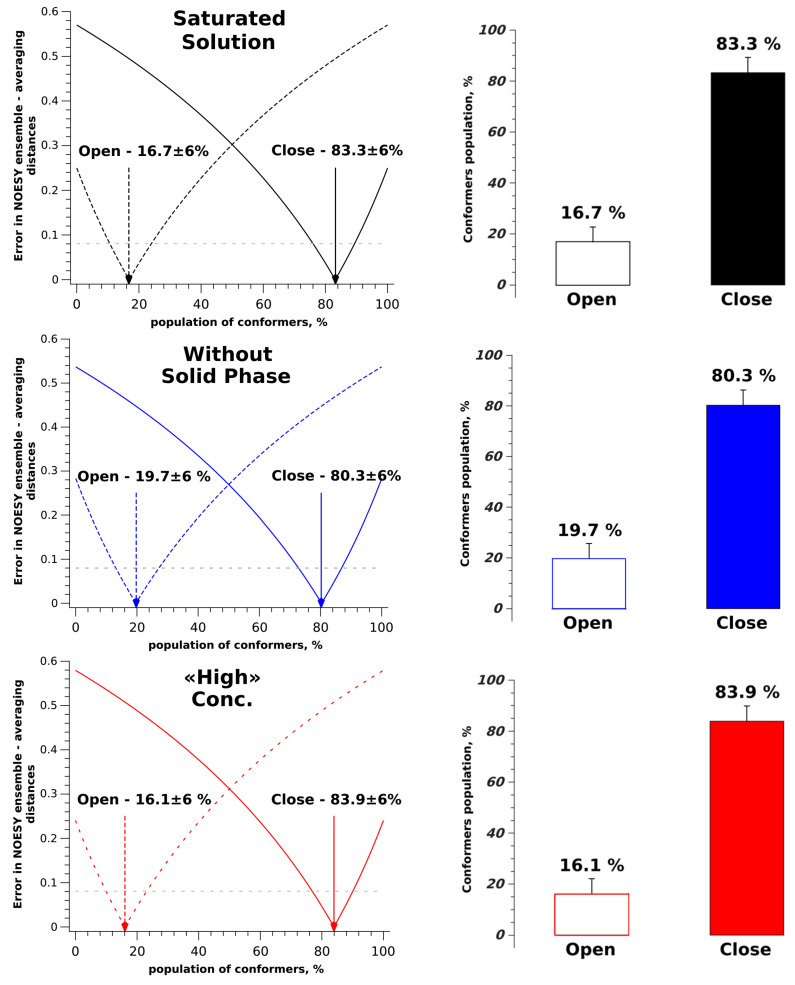

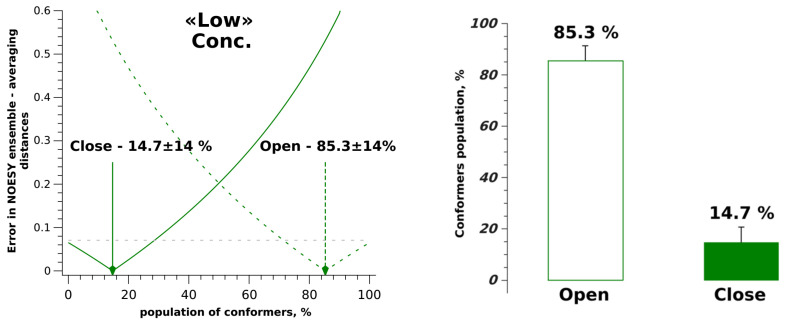
(**Left column**)—graphs of the difference between calculated and experimental values of the H12b-H14/18 distance as a function of the conformer population. The gray dashed line indicates the error limit in determining the conformer-defined distance from NOESY data. (**Right column**)—bar charts showing the distribution of «open» and «closed» conformers obtained from NOESY data and the H12b-H14/18 distance as conformer-defined.

For all the studied systems, the cross-relaxation rate σ_0_ remains unchanged, as the distance between the CH_2_ protons (H12a-H12b) does not change significantly across the conformers. However, the value of σ_exp_ for «low» concentration differs markedly for the remaining systems. This trend is reflected in the experimentally determined inter-nuclear distances (r_exp_). The obtained experimental distances were used to calculate the proportions of conformer groups within a two-position exchange model [48,59]. A graphical approach was used to visualize the model and determine the error in the proportions of the conformer groups (see Figure 4), as previously described [60].

The results of the calculation of the proportions of «open» and «closed» conformer groups show that in saturated solution, system without the solid phase and «high» concentration solution, the desired values remain unchanged within the error, with the proportion of «open» conformers at 17% and «closed» conformers at 83%. This indicates that for BCL, the presence or absence of the solid phase does not significantly influence the observation of specific molecular conformations in solution. Instead, solvation processes play a crucial role, especially when the concentration of BCL in CDCl_3_ is significantly reduced, as in the case of a system with «low» concentration. This shift suggests that solvent interactions play a crucial role in stabilizing specific conformers. These observations are consistent with previous studies that reported solvent polarity influencing the conformer ratio of BCL, with non-polar solvents favoring the «closed» form and polar solvents promoting the «open» form [28]. Figure 4 shows the transition from «high» to «low» concentration results in a sharp change in conformation from «closed»—83.9% to «open»—85.3%. This transition occurs due to the addition of 0.15 mL of solvent. At the same time, the lack of changes in the distribution of conformers when transitioning from a system without the solid phase to a «high» concentration system suggests that there is a broad concentration range where BCL molecules are stabilized in the «closed» conformation.

Our approach to analyzing the obtained results and identifying patterns in the stabilization of «open» and «closed» BCL conformers in CDCl_3_ solutions of varying concentrations involved a series of additional experiments. ROESY spectra were recorded for a saturated solution of BCL in CDCl_3_ (see Figure S3). The joint NOESY and ROESY spectral data analysis, a well-known method for characterizing magnetization transfer types, detecting spin diffusion effects, and intermolecular interactions, was conducted with utmost care [61]. The comparison of NOESY and ROESY spectra, which revealed all characteristic cross-peaks in phase, further instills confidence in the direct magnetization transfer between the nuclei of interest and the absence of spin diffusion effects. The interactions of interest (H12a-H12b and H12b-H14/18) predominantly appear in the 1D NOESY spectrum (see Figure S4), further supporting the relevance of the selected reference and conformation-dependent distances.

The ^1^H NMR spectra, recorded at temperatures of 20, 30, 40, and 50 °C (see Figure S5), underwent a comprehensive analysis. While such an analysis can provide a deeper understanding of conformational characteristics for small molecules [62,63,64], in our case, no significant changes were observed that would indicate exchange processes or conformational changes with increasing temperature. However, to identify possible dimerization effects in the saturated solution, which could also drive conformational changes, we compared DOSY spectra obtained for the saturated solution and the «low» concentration solution (see Figure S6). The data were analyzed in detail, and it was found that in the saturated solution, all proton signals of the molecules allow for the determination of the self-diffusion coefficient as D_BCL_ = (5.52 ± 0.33) × 10^−10^ s^−1^. In contrast, the residual solvent proton signal gives D_CDCl3_ = 1.82 × 10^−10^ s^−1^. For the «low» concentration solution, the values are (5.72 ± 0.36) × 10^−10^ s^−1^ and 1.61 × 10^−10^ s^−1^, respectively. A direct comparison of values obtained for BCL is not always straightforward; however, qualitatively, it demonstrates a decrease in magnitude when transitioning from a low-concentration solution to a saturated solution, which is a reliable indicator of association processes. For the quantitative analysis of the obtained data, a graphical approach was employed to examine the experimentally determined D_BCL_ values as a function of the molecular weight of the complex. D_CDCl3_ values were used as a reference [65]. It is well known that the ratio of D_BCL_/D_CDCl3_ lies within the square and cubic root values of the molecular mass ratio of the reference compound (CDCl_3_) and the formed associate [66,67,68,69,70,71] (see Equation (1)):

(1) $$\sqrt{\frac{M_{CDCL3}}{M_{BCL}}}\geq \frac{D_{BCL}}{D_{CDCL3}}\geq \sqrt[{3}]{\frac{M_{CDCL3}}{M_{BCL}}}$$

where M_CDCL3_ and M_BCL_ are the molecular weights of the reference compound and the BCL-based associate, and D_BCL_ and D_CDCL3_ are the diffusion coefficients of the reference compound and the formed associate, respectively.

**Figure 5:**
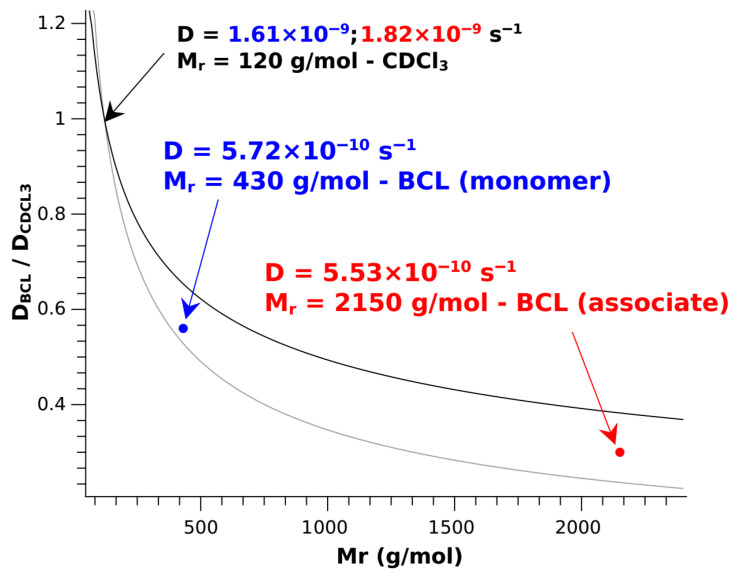
Graphical analysis of self-diffusion coefficients versus molecular weight from DOSY NMR spectroscopy. The blue dot represents the monomeric structure of BCL, the red dot represents the structure of an associate of five BCL molecules, and the black dot represents the reference compound (CDCl_3_).

In accordance with Equation (1) and the square and cubic root values of the molecular mass ratios, the theoretical range of D values for the expected associates was determined (see Figure 5—gray and black lines).

As shown in Figure 5, the obtained D values for the two investigated systems fall within the range of theoretical values, underscoring the significance of our findings. Moreover, it can be stated that in the saturated solution with a solid phase, the predominant associates consist of 4–6 BCL molecules (with a molecular weight over 2000 g/mol). In contrast, in the low-concentration solution, predominantly monomeric BCL molecules are present. These results are in good agreement with the spatial structure data for BCL in the studied systems. Apparently, when existing as associates (at high concentrations and in a saturated solution), BCL forms a «closed» conformation. However, when transitioning to low-concentration solutions, BCL molecules, which exist in a monomeric form, adopt an «open» conformation. Thus, the driving force behind the conformational changes in BCL is not the presence or absence of a solid phase but rather the associative processes, accompanied by the formation and dissociation of various non-covalent interactions. These findings are presented and definitively proven for BCL for the first time, significantly complementing the overall mechanistic picture of previously conducted studies [28].

These unique findings not only underscore the validity of previous notions regarding the stabilization of the spatial structure of BCL molecules through a complex system of intra- and intermolecular non-covalent interactions [24,33], but also provide a strong confirmation. It has been previously noted that CDCl_3_ molecules may potentially disrupt weak π⋯π interactions and influence the redistribution of intra- and intermolecular hydrogen bonds, causing the «opening» of the structure as the concentration decreases.

Previous studies [33,72] show that association processes can stabilize BCL conformers. They suggest that intramolecular and intermolecular interactions influence the formation of these conformers. This study supports the idea that solvents indirectly influence conformational preferences by influencing the ability to associate and thereby shifting conformational equilibria.

In line with this hypothesis, a detailed analysis was conducted on the energetic and geometric characteristics of noncovalent interactions, both in the monomeric structures of BCL and in the associated structures (using dimers as a model). The study found that dimers of «closed» BCL conformers have the lowest interaction energy at −134.47 kJ/mol, compared to −102.02 and −109.08 kJ/mol for dimers of «open» conformers. The stabilization of the «closed» BCL dimers is attributed to the redistribution of multiple intra- and intermolecular interactions, as well as other weak noncovalent interactions, which are primarily responsible for stabilizing BCL associates. The study [33] reveals that «closed» BCL conformers exhibit a wider range of noncovalent interactions due to the close arrangement of their cycles, including N–H···O(H), C–H···O(C), and others. In contrast, «open» structures primarily exhibit interactions such as N–H···O(H) and C–H···O(C).

In the present work, based on DOSY data, we obtained explicit quantitative confirmation of the previously suggested hypotheses, which were based on quantum-chemical calculations and calculations of solvation and deformation energies of the BCL structure in the presence of CDCl_3_ [28]. The trends in the conformational changes in BCL in CDCl_3_ are similar to those previously observed for the BCL–DMSO-*d*_6_ system [31]. Thus, the results of the present work firm and reassuring conclusion to a series of previously conducted studies, offering a definitive experimental interpretation of the patterns of BCL spatial structure stabilization. Previous research has suggested that solvents affect conformational preferences. This study confirmed that idea, demonstrating that the solvent’s influence is indirect, impacting the ability to form specific associates.

## 3. Materials and Methods

The study used commercially available compounds from BLD Pharmatech (Hyderabad, India) and Sigma-Aldrich (St. Louis, MO, USA): Bicalutamide (BCL) (*N*-[4-cyano-3-(trifluoromethyl)phenyl]-3-[(4-fluoro-phenyl)sulfinyl]-2-hydroxy-2-methyl-propanamide; CAS No: 90357-06-5) and deuterated chloroform (CDCl_3_; chloroform-d_1_; CAS No: 865-49-6). The substances were used without further purification. NMR data were acquired using a Bruker Avance III 500 MHz spectrometer and a Bruker TBI probe (5 mm, Billerica, MA, USA). Throughout all NMR experiments, the temperature was maintained at a constant value (297 K) using a BVT 3000 (Bruker, Billerica, MA, USA)and BCU 05 (Bruker, Billerica, MA, USA) control unit. In this study, NMR spectra were recorded for four systems: a saturated solution (with a solid phase) of BCL in CDCl_3_, a solution without the solid phase, a solution at «high» concentration, and a solution at «low» concentration.

To prepare a saturated solution, a BCL sample (5.5 mg) was placed in an NMR tube (Wilmad, Vineland, NJ, USA), followed by the addition of CDCl_3_ (1 mL). The tube was immediately sealed to prevent the loss of volatile solvent. The sealed tube was placed in a laboratory air thermostat and incubated for 24 h at 297 K. After incubation, a solid phase was observed, and the tube was immediately transferred to the NMR spectrometer probe (Bruker, Billerica, MA, USA), where the temperature was maintained at 297 K, and NMR spectra were recorded. Following the analysis of the saturated solution, the NMR tube was removed from the spectrometer, placed in the thermostat, and opened. A 0.6 mL aliquot of the liquid phase was transferred to a new NMR tube, with filtration performed using a syringe filter equipped with a nylon membrane (NY) with a pore diameter of 0.45 μm. To remove any residual crystalline BCL phase and to investigate the solution without the solid phase, an additional 20 μL of solvent was added to the tube. The tube was sealed and thermally stabilized; the spectra were then recorded.

To prepare a solution at «high» concentration and conduct further analysis of the conformational behavior of BCL in CDCl_3_, 0.15 mL of solvent was added to the previously prepared sample, and for a solution at «low» concentration, another 0.15 mL of solvent was added.

^1^H NMR spectra were recorded using the «zg» pulse sequence implemented in the TopSpin 3.6.1 software package. The spectral range was 14.3 ppm, with 32 scans, a relaxation delay of 1 s, and 32,768 FID data points. NOESY 2D spectra were recorded using the «noesygpphpp» pulse sequence, with a spectral range of 14.3 ppm in both dimensions, 16 scans (2 cycles (TD) of 8 scans (NS)). The mixing time (τ_m_) for the pulse sequence was 0.9, 0.8, 0.6, and 0.4 s. Detailed information about the structure of BCL molecules, as well as calculated data for the likely «closed» and «open» conformers, can be found in previous studies [33,73]. Details of sample preparation and additional experiments (DOSY, 1D NOESY, ROESY and VT-NMR (^1^H) data) can be found in the Supporting Information Section. Data were obtained using density functional theory with the Gabedit V. 2.5.2 software package [74].

## 4. Conclusions

Based on the data from NMR spectroscopy, a quantitative analysis of the proportions of «open» and «closed» conformer groups for four BCL systems in CDCl_3_ at different concentrations, as well as in the presence and absence of a solid phase, was conducted. Despite the extremely low solubility of BCL in CDCl_3_ for NMR analysis, it was established that conformational changes in BCL molecules are not dependent on the presence or absence of the solid phase. The primary driving force behind these changes is solvation effects, which result from concentration variations and disrupt multiple non-covalent interactions, causing the «opening» of the structure.

Conformational transitions occur within a narrow concentration range. As the concentration of the substance increases, the «closed» structure of BCL, observed in the most stable polymorphic form, is stabilized. The results of this study extend the understanding of the conformational evolution of small molecules in solution by showing, using bicalutamide as an example, that solvation effects, rather than the solid phase, primarily drive conformational changes.

## Figures and Tables

**Table 1:** Cross-relaxation rates, experimental and calculated distances for the four studied BCL systems in CDCl_3_.

System	σ_exp×10_^−3^, s^−1^	σ_0×10_^−1^, s^−1^	r_exp_, Å	r_Close_, Å	r_Open_, Å	r_0_, Å
Saturated solution	1.87 ± 0.21	2.52 ± 0.99	4.03 ± 0.08	4.28 ± 0.18	3.46 ± 0.49	1.78 ± 0.01
Without the solid phase	1.96 ± 0.11	2.51 ± 0.11	3.99 ± 0.08			
«High» concentration	1.85 ± 0.23	2.47 ± 0.14	4.04 ± 0.08			
«Low» concentration	4.16 ± 0.37	2.51 ± 0.16	3.53 ± 0.07			

## Data Availability

The original contributions presented in this study are included in the article and Supplementary Materials. Further inquiries can be directed to the corresponding author.

## Supplementary Materials

The following supporting information can be downloaded at https://www.mdpi.com/article/10.3390/molecules30224479/s1: Figure S1: Fragment of the ^1^H NMR spectra for a saturated solution of BCL in CDCl_3_, showing the OH group signal of BCL molecules at different time intervals; Figure S2: Dependence of averaged integral intensities on mixing time for the reference and conformer-defined distances from NOESY spectra analysis; Table S1: Values of integral intensities, cross-relaxation rates, and errors from 2D NOESY spectra for the four studied systems, BCL in CDCl_3_; Figure S3: Comparison of NOESY and ROESY spectra of a saturated solution of BCL in CDCl_3_; Figure S4: 1D NOESY spectrum of a saturated solution of BCL in CDCl_3_ with H12b selected as the “irradiated” signal; Figure S5: ^1^H NMR spectra of the saturated solution of BCL in CDCl_3_ at different temperatures; Figure S6: DOSY NMR spectra of the saturated and “low” concentration solutions of BCL in CDCl_3_.
