# Supplementary material for: Conformational Evolution of Bicalutamide in Chloroform: A Comprehensive NMR Study
Source: Molecules. 2025 Nov 20;30(22):4479. doi: 10.3390/molecules30224479 (PMC12655020; doi:10.3390/molecules30224479)
Supplement: Supplementary file 1 [file molecules-30-04479-s001.zip › molecules-3963568-supplementary.pdf]

molecules

MDPI

SUPPLEMENTARY INFORMATION

For additional experiments, the results of which were crucial in identifying the stabilization patterns of the spatial structure of BCL in chloroform solution, a sample of a saturated solution was used (with a solid, undissolved phase at the bottom of the NMR tube). The sample mass was 5.5 mg, and the solvent volume was 1 mL. To prepare the "low" concentration sample, the liquid phase was withdrawn from the NMR tube and then transferred to a new one using a syringe filter equipped with a nylon membrane (NY) with a pore diameter of 0.45 μm. Additional experiments were first performed for the saturated solution sample, and then for the "low" concentration sample obtained by dilution. The experiments were also conducted using the Bruker Avance III 500 MHz spectrometer, and temperature control was maintained using the BVT 3000 and BCU 05 blocks.

^{1}H-^{1}H ROESY spectra were recorded using the pulse program "roesyph," with a spectral range of 14.08 ppm along F1 and F2 axes, 128 scans, relaxation delay of 3 seconds, mixing time of 0.3 seconds, FID data points along F1 and F2 axes of 128 and 2048, respectively, and experiment duration of 16.6 hours. 1D NOESY spectra were obtained using the pulse program "selnogpzs.2," with a spectral range of 14.3 ppm, 1024 scans, relaxation delay of 4.7 seconds, mixing time of 0.9 seconds, FID data points of 32768, and experiment duration of 2.2 hours. ^{1}H NMR spectra were recorded using the "zg" program at 293, 303, 313, and 323 K. After setting each temperature, the sample was equilibrated in the NMR spectrometer's probe for 1 hour. During the spectra registration, the spectral range was 11.5 ppm, 32 scans were performed, the relaxation delay was 1 second, FID points were 32768, and the experiment duration was 2 minutes.

2D DOSY spectra were recorded using the "ledbpgp2s" pulse sequence (stimulated echo and longitudinal eddy current delay using bipolar gradient pulses). The gradient trajectory contained 16 points; the diffusion time Δ was set to 0.05 seconds, and the pulse duration δ = 2.8 ms. To calculate the diffusion coefficient values (D_{exp}), experimental diffusion decay curves were obtained, which represent the dependence of the relative integral intensity (I/I_0) on the gradient field strength on a logarithmic scale. The obtained curves were approximated using a mathematical model that includes experimental parameters and the sought diffusion coefficient (see equation S1). The calculation was performed in the NMR spectrometer software package TopSpin 3.6.1:

$$\mathrm{I} = \mathrm{I}[0] \times \exp(-\mathrm{D}_{\mathrm{exp}} \times SQR(2 \times PI \times gamma \times Gi \times LD) \times (BD - LD/3) \times 10^4) \qquad \text{S1}$$

where gamma is the gyromagnetic ratio of the investigated nuclei ($4.258 \cdot 10^3$ Hz/G), Gi is the power of the gradient pulse, the big delta (BD) is 49.9 ms, and the little delta (LD) is 1.4 ms.

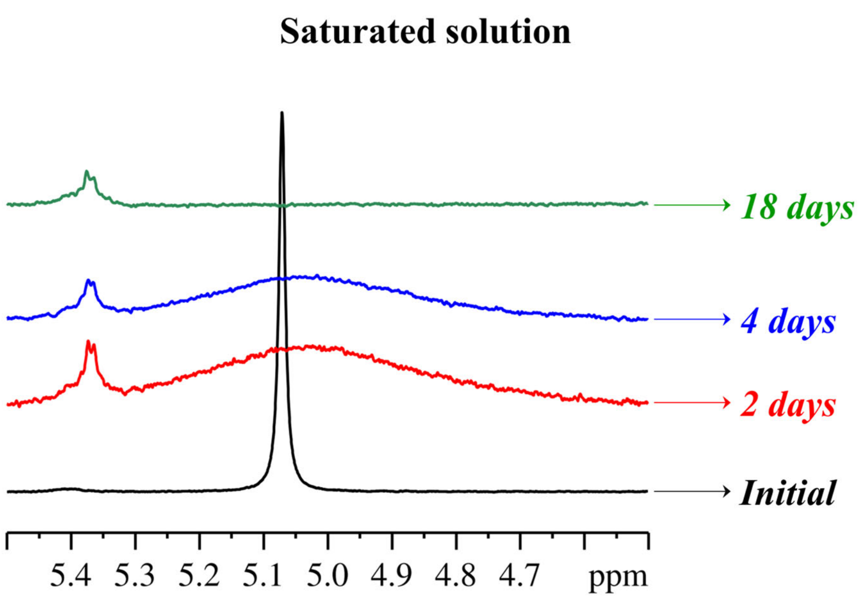

Figure S1. Fragment of the ^{1}H NMR spectra for saturated solution of BCL in $CDCl_3$, showing the OH group signal of BCL molecules, recorded immediately after sample preparation (black line), after 2 days (red line), after 4 days (blue line), and after 18 days (green line)

Saturated solution

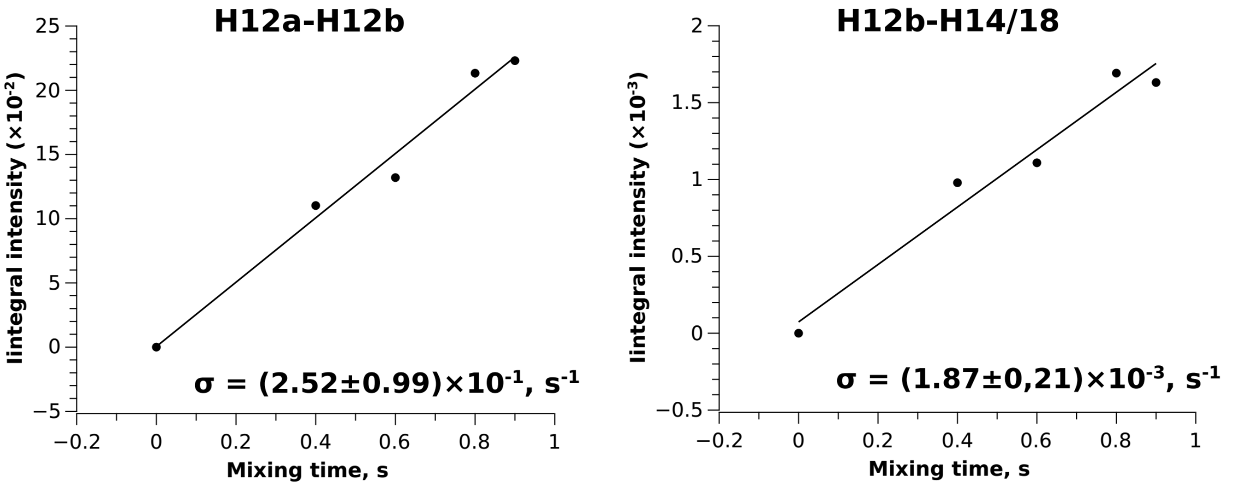

Without solid phase

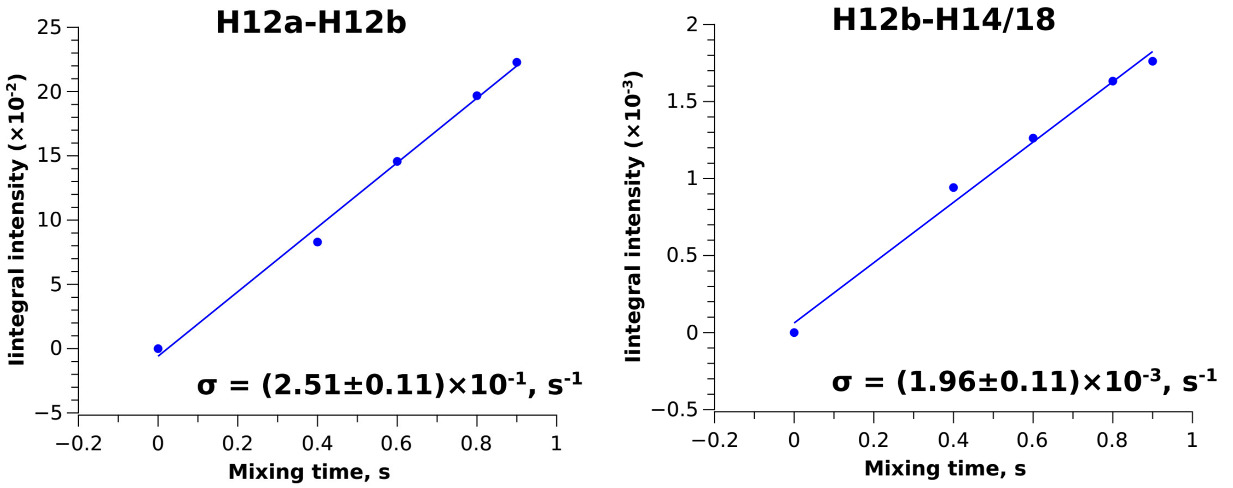

«High» concentration

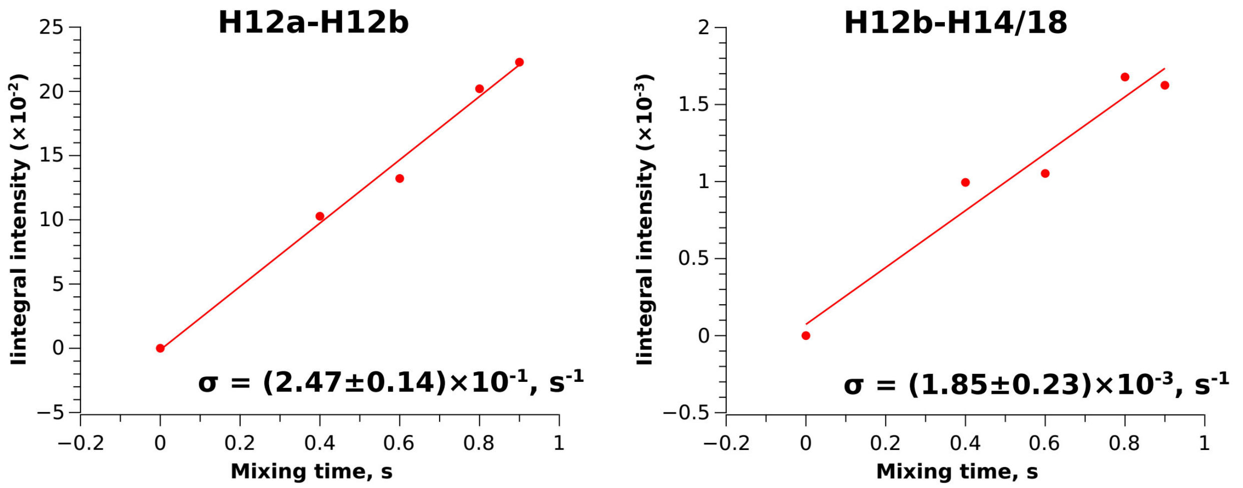

«Low» concentration

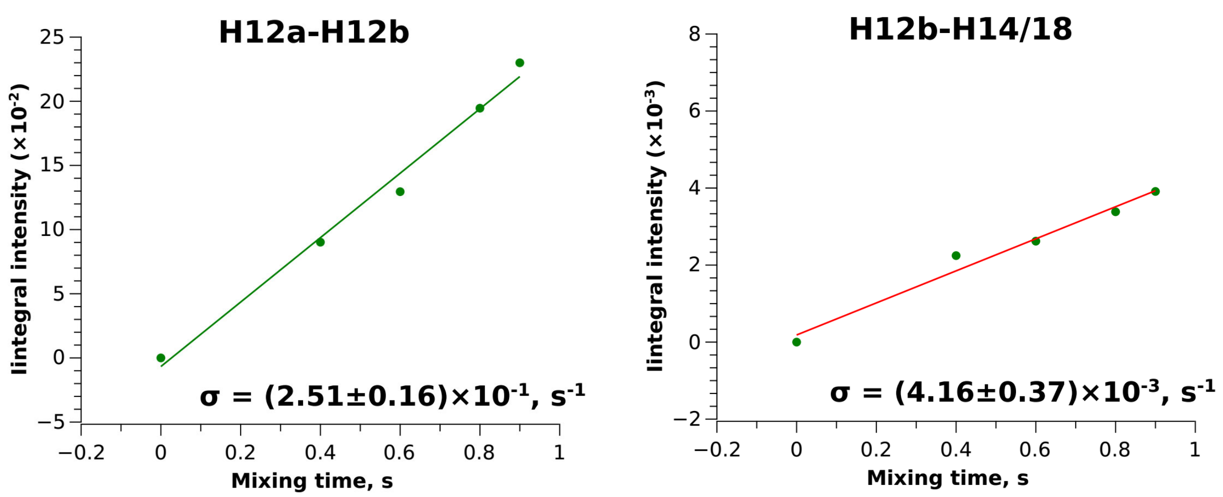

Figure S2. Dependence of averaged integral intensities on mixing time for the reference (left column) and conformer-defined (right column) distances, obtained from the analysis of NOESY spectra for the four studied systems

Table S1. Values of integral intensities of cross-peaks, cross-relaxation rates, and their errors obtained from 2D NOESY spectra for the four studied systems, BCL in $CDCl_3$.

System	Distance	Mixing time	Integral Intensity	Cross relaxation	Error
Saturated solution	H12a-H12b	0.4	0.1102	2.52×10^{-1}	$\pm0.99\times10^{-1}$
		0.6	0.1320		
		0.8	0.2133		
		0.9	0.2230		
		0	0		
	H12b-H14/18	0.4	0.0010	1.87×10^{-3}	$\pm0.21\times10^{-3}$
		0.6	0.0011		
		0.8	0.0017		
		0.9	0.0016		
		0	0		
Without solid phase	H12a-H12b	0.4	0.0829	2.51×10^{-1}	$\pm0.11\times10^{-1}$
		0.6	0.1457		
		0.8	0.1968		
		0.9	0.2228		
		0	0		
	H12b-H14/18	0.4	0.0009	1.96×10^{-3}	$\pm0.11\times10^{-3}$
		0.6	0.0013		
		0.8	0.0016		
		0.9	0.0018		
		0	0		
«High» concentration	H12a-H12b	0.4	0.1028	2.47×10^{-1}	$\pm0.14\times10^{-1}$
		0.6	0.1322		
		0.8	0.2020		
		0.9	0.2227		

		0	0		
	H12b-H14/18	0.4	0.0010	1.85×10^{-3}	±0.23×10^{-3}
		0.6	0.0011		
		0.8	0.0017		
		0.9	0.0016		
		0	0		
«Low» concentration	H12a-H12b	0.4	0.0902	2.51×10^{-1}	±0.16×10^{-1}
		0.6	0.1295		
		0.8	0.1946		
		0.9	0.2300		
		0	0		
	H12b-H14/18	0.4	0.0022	4.16×10^{-3}	±0.37×10^{-3}
		0.6	0.0026		
		0.8	0.0034		
		0.9	0.0039		
		0	0		

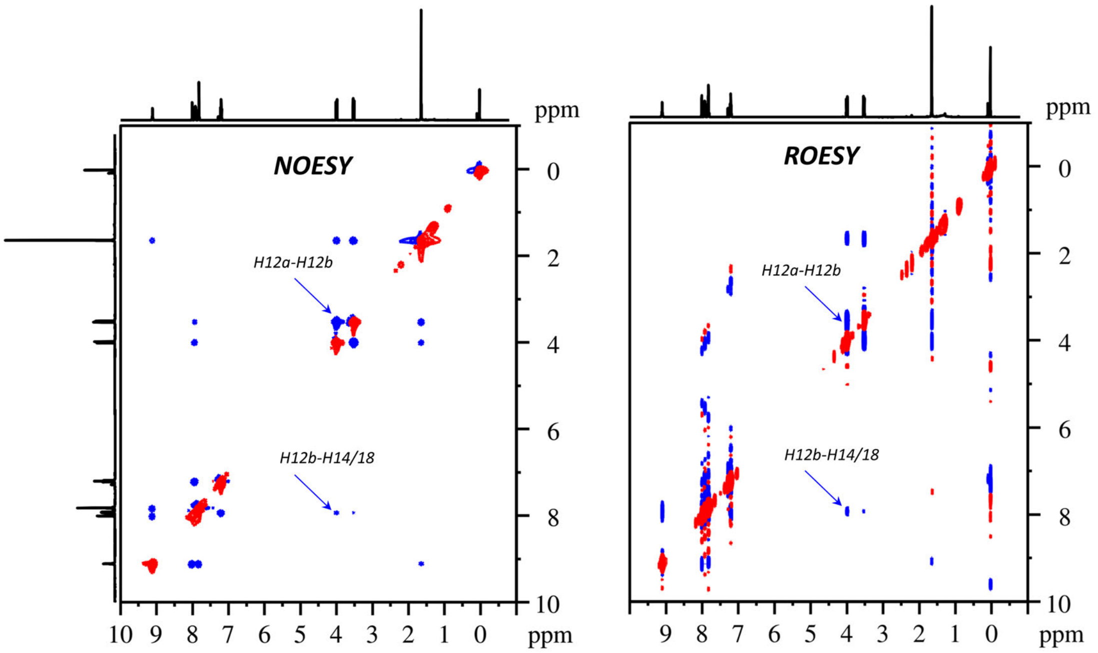

Figure S3. Comparison of NOESY and ROESY spectra of a saturated solution of BCL in $CDCl_3$.

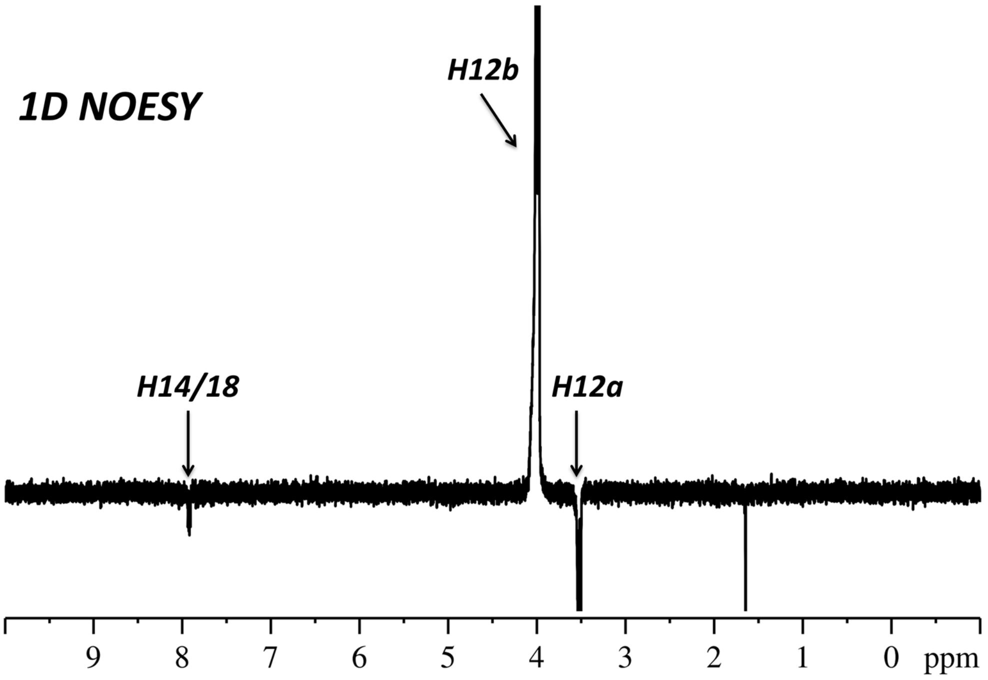

Figure S4. 1D NOESY spectrum of a saturated solution of BCL in $CDCl_3$. The proton signal H12b was selected as the "irradiated" one.

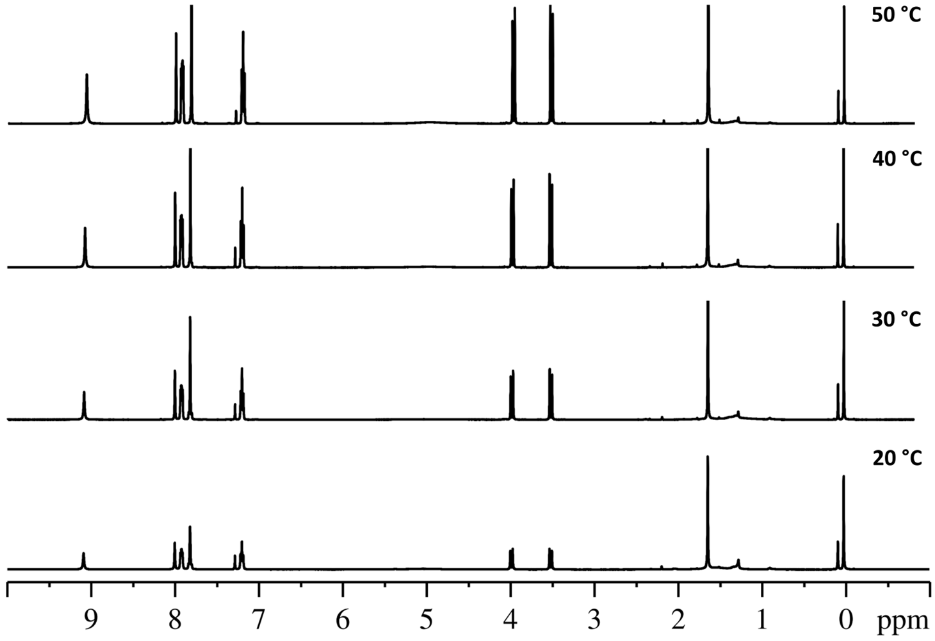

Figure S5. ^{1}H NMR spectra of the saturated solution of BCL in $CDCl_3$ recorded at 20, 30, 40, and 50 °C.

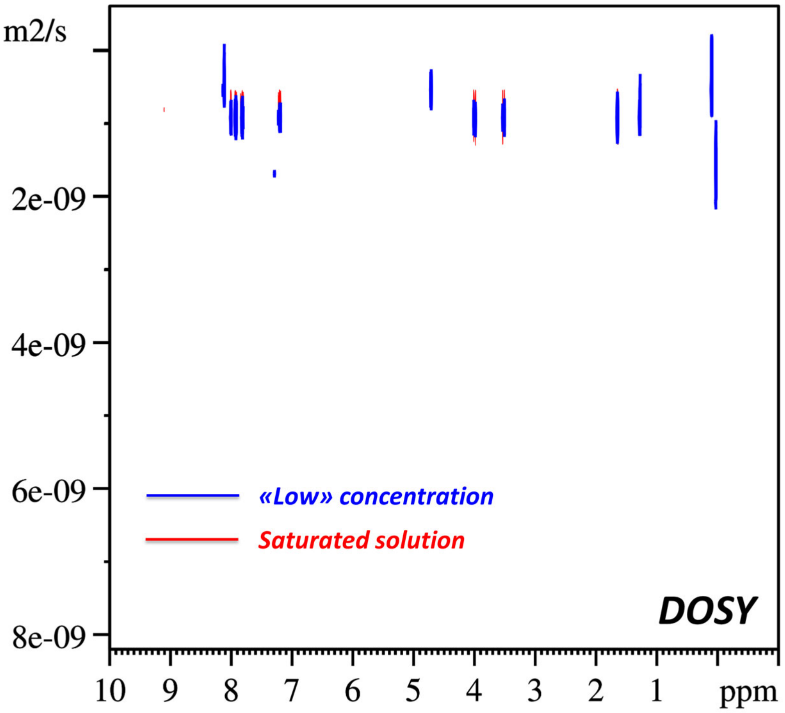

Figure S6. DOSY NMR spectra of the saturated solution (red) and the "low" concentration solution (blue) of BCL in $CDCl_3$.
